# A Case‐Control Study on the Association Between *MMP2* and *MMP9* Genetic Polymorphisms and Breast Cancer

**DOI:** 10.1002/hsr2.71123

**Published:** 2025-07-28

**Authors:** Pulak Chowdhury, Md. Abdul Aziz, Tahmina Akter, Mohammad Safiqul Islam, Md. Shahid Sarwar

**Affiliations:** ^1^ Department of Pharmacy Noakhali Science and Technology University Noakhali Bangladesh

**Keywords:** breast cancer, *MMP2*, *MMP9*, polymorphism, rs2285053, rs3787268

## Abstract

**Background and Aims:**

Matrix metalloproteinase 2 (MMP2) and MMP9 are associated with the degradation of type IV collagen, leading to invasion and metastasis. Polymorphisms in these genes could influence their biological activities and contribute to cancer development and progression. This study evaluated the relationship between *MMP2* (rs2285053) and *MMP9* (rs3787268) gene polymorphisms and the susceptibility to breast cancer in Bangladeshi breast cancer patients.

**Methods:**

This case‐control study included 105 females diagnosed with breast cancer and 108 healthy controls. Following DNA extraction from the blood samples, genotyping was carried out by tetra‐primer amplification refractory mutation system‐polymerase chain (ARMS‐PCR) reaction and gel electrophoresis.

**Results:**

In the case of rs2285053 polymorphism, CT genotype (OR = 2.12, 95% CI = 1.03–4.36), dominant model (OR = 2.21, 95% CI = 1.08–4.52), overdominant model (OR = 2.10, 95% CI = 1.02–4.31), and alleles (OR = 2.13, CI = 1.08–4.18) were significantly associated with an increased breast cancer risk. For rs3787268 polymorphism, additive model 1 (OR = 0.20, 95% CI = 0.05–0.78), additive model 2 (OR = 0.23, 95% CI = 0.06–0.86), and dominant model (OR = 0.22, CI = 0.06–0.81) significantly decreased breast cancer risk in this population.

**Conclusion:**

Our results conclude that *MMP2* (rs2285053) is associated with an increased risk of breast cancer, while *MMP9* (rs3787268) polymorphisms may be correlated with a reduced risk of breast cancer in Bangladeshi females. Future studies are warranted to validate our findings in other populations.

## 1 Background

Breast cancer is the most frequent cancer in females and the most prevalent malignancy globally [1]. In 2020, more than two million females were identified with breast cancer, about 11.7% of total cancer cases worldwide. It is also considered the fifth leading cause of death worldwide, with ~684,996 deaths [2]. The incidence of breast cancer is on the rise in South Asian countries, which is concerning. According to the Global Cancer Observatory (GLOBOCAN) 2020, in Bangladesh, the number of new cases of breast cancer was 8.3% for both sexes, where female breast cancer is ranked in first position (19.0%) in terms of incidence and fourth in terms of mortality (6.2%) [3].

It is important to note that genetic and nongenetic causes lead to breast cancer development. Menstrual and reproductive history, body mass index, alcohol consumption, and level of physical exercise are all examples of nongenetic risk factors [4]. Even though the exact cause of breast cancer is unknown, it is primarily assumed that genetic factors significantly influence it. Genetic mutations have been identified in recent decades as the risk factors for breast cancer, and the genetic diversity in particular genes is responsible for around 5% to 10% of all breast cancer cases [5, 6]. Genome‐wide association studies have identified several single‐nucleotide polymorphisms (SNPs) of numerous genes that are connected with the development of this cancer [7].

Matrix metalloproteinases or MMPs are a family of multifunctional Zn^2+^‐dependent endopeptidases that participate in the degradation of extracellular matrix proteins and glycoproteins [8, 9], cytokines [10], membrane receptors [11], growth factors [12], and basement membrane barriers, as well as serving an essential role in the separation of tumor cells from the surrounding normal tissues [13, 14, 15]. Human tissue expresses at least 23 of the 28 forms of MMPs in vertebrates [16, 17]. MMPs can be categorized into five categories [17], and MMP2 and MMP9 belong to one of these categories, containing three fibronectin‐like inserts in the catalytic domain [18]. The *MMP2* gene, which codes for gelatinase A, is located on chromosome 16, while the *MMP9* gene, which encodes gelatinase B, is found on chromosome 20 [19]. They belong to collagenase IV and play critical roles in tumor cell differentiation, angiogenic activity, invasion, and metastatic processes [20]. Several studies have indicated that *MMP2/MMP9* is an important prognostic factor for various cancer types. Increased *MMP2/MMP9* levels in the blood were associated with a considerably reduced chance of survival for females with breast cancer [21, 22, 23]. Mammary tumors that are invasive and have a poor prognosis are related to overexpression of *MMP2* or *MMP9* [22, 23, 24]. In addition to breast carcinomas, several other types of cancer, such as oral cancer [25], retinoblastoma [26], bladder cancer [27], and ovarian epithelial cancer [28], have been associated with *MMP2* and *MMP9* overexpression.

rs2285053 is located at position −735 in the promoter of *MMP2* and indicates a transition from the common allele C to T [19]. This genetic polymorphism was reported to be linked with cancer development through the alteration of protein expression levels by affecting gene transcriptional activities [20]. On the other hand, the chromosomal position of rs3787268 is at Chr20:44075138 in intron 8 of *MMP9* and indicates a transition from the common allele G to A [29]. Several studies suggested that the rs3787268 polymorphism was associated with an increased risk of breast cancer in the Chinese Han population [30] and the Hispanic population [31], while Fu and colleagues showed that a significant association was found between *MMP9* rs3787268 GA + AA genotypes and poor disease‐free survival in Chinese breast cancer patients, but did not significantly increase the risk [29].

To the best of our knowledge, no prior study has been conducted to find the association of *MMP2* and *MMP9* genetic polymorphisms with breast cancer risk in Bangladeshis. Therefore, our study aimed to investigate the association between *MMP2* (rs2285053) and *MMP9* (rs3787268) gene polymorphisms with breast cancer in Bangladeshi females.

## 2 Methods

### 2.1 Study Design and Selection of Participants

The current case‐control study was conducted on a total of 105 individuals who had been diagnosed with breast cancer and had been recruited between 2016 and 2018 as cases from the National Institute of Cancer Research and Hospital in Dhaka, Bangladesh (NICRH/Ethics/2019/446). The study controls were 108 healthy individuals from around the country. Informed consent was obtained from all study participants before conducting the study. Ethical permission was obtained from the ethical committee of the Noakhali Science and Technology University. The reporting of this study followed the Strengthening the Reporting of Observational Studies in Epidemiology (STROBE) guidelines designed for case‐control studies [32].

### 2.2 Collection and Storage of Blood, Isolation, and Quantification of Genomic DNA

Peripheral blood samples (about 3 mL) were obtained from all study participants, including both cases and controls, and stored at −80°C until DNA was extracted using EDTA (ethylenediaminetetraacetic acid). The “FavorPrep” DNA extraction mini kit was used following the protocol book provided with the product to extract genomic DNA. The microvolume spectrophotometer (Genova Nano, Jenway) was used to determine DNA concentration. The purity of genomic DNA was determined by comparing the 260 and 280 nm absorption ratios.

### 2.3 Design of Primer and Genotyping

The tetra‐primer amplification refractory mutation system polymerase chain reaction (ARMS‐PCR) method was adopted to complete the SNP genotyping process following the protocols published previously [33]. The online programming tool Primer 1 was used for the primer design process. The desired allele was amplified using four primers: forward outer, forward inner, reverse outer, and reverse inner (Table 1). Primers were intermixed with nuclease‐free water, MgCl_2_, and EmeraldAmp GT PCR Master Mix at a specified concentration to formulate a PCR premix. For a 120 μL PCR master mix solution (12 samples; 10 μL/reaction), the volume of outer primers (forward outer and reverse outer) was 1.5 μL, and inner primers (forward inner and reverse inner) were 3 μL. To execute the PCR, the DNA sample was added to the premix (10 μL) at the desired concentration. After that, the PCR products were analyzed using gel electrophoresis on an agarose gel with a 1% concentration to confirm the DNA bands corresponding to specific alleles stained with ethidium bromide.

**Table 1:** Primer sequences used in the tetra‐primer ARMS‐PCR method.

SNPs	Primers	Sequence (5′–3′)	Allele	Amplicon size (bp)
rs2285053	FI	TATCTCATCCTGTGACCGAGAATGCGGCCC	C	
	RI	ACCTGCTGGGCTGCACTCCCAGGCGA	T	CC—146
	FO	TGCCATGGCACTGGTGGGTGCTTCCTTT		CT—243
	RO	GGAAATAGAGCAGTCAGGGGCCCCGCG		TT—333
rs3787268	FI	GGCCATAGAGGATGTCGCTTAAACCA	A	
	RI	AACCACAGGACTTTCTTCTTCTTCTTTGTC	G	AA—165
	FO	GACTTCAAAAGCCAGTCTACTCTGGGC		AG—330
	RO	TCTTCCTATTCCTGCATACCTCTGTACCC		GG—439

### 2.4 Process for Validating the ARMS‐PCR Method

**Figure 1:**
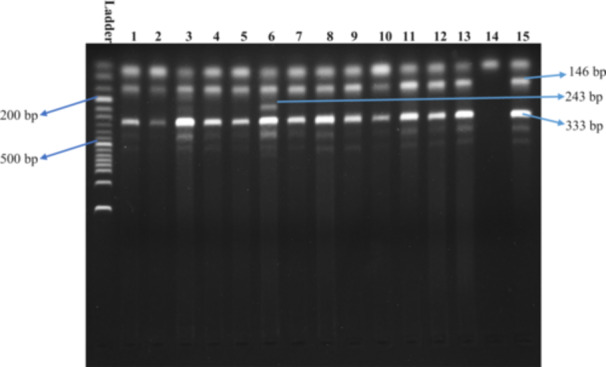
PCR amplification bands of CC, CT, and TT genotypes for SNP rs2285053 and product sizes were 146 bp for C allele, 243 bp for T allele, and 333 bp for the control band. The first lane represents a 50 bp DNA ladder; lanes 1–5, 7–13, and 15 indicate CC genotype; lane 6 indicates CT genotype; and lane 14 is blank.

**Figure 2:**
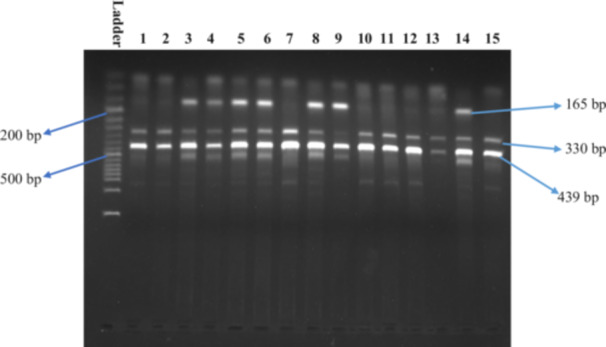
PCR amplification bands of AA, AG, and GG genotypes for SNP rs3787268 and product sizes were 165 bp for A allele, 330 bp for G allele, and 439 bp for the control band. The first lane represents a 50 bp DNA ladder; lanes 1, 2, 7, 10–13, and 15 indicate GG genotype; lanes 3–6, 8, and 14 indicate AG genotype; and lane 9 indicates AA genotype.

Method validation was undertaken to select an appropriate annealing temperature for the specified SNPs to obtain the requisite DNA fragments. Primer melting temperatures were determined manually. We consecutively selected 68°C and 60°C temperatures as our desired annealing temperatures for *MMP2* rs2285053 and *MMP9* rs3787268. For rs2285053, at 68°C temperature, we detected 333 bp, 243 bp, 146 bp fragments (Figure 1), whereas for rs3787268 SNP, at 51°C temperature, we detected 439 bp, 330 bp, 165 bp fragments (Figure 2). Table 2 shows the details of the specific PCR conditions for the rs2285053 and rs3787268 SNPs and their fragment size.

**Table 2:** PCR conditions for rs2285053 and rs3787268 with fragment size.

SNPs	PCR condition	No. of PCR cycles	PCR product size (bp)	Genotype
rs2285053	95°C for 5 min	35 cycles		
	95°C for 1 min		NH: 243, 333	CC
	68°C for 45 s		HE: 146, 243, 333	CT
	72°C for 1 min		MH: 146, 333	TT
	72°C for 10 min			
rs3787268	95°C for 5 min	35 cycles		
	95°C for 1 min		NH: 330, 439	AA
	60°C for 45 s		HE: 165, 330, 439	AG
	72°C for 1 min		MH: 165, 439	GG
	72°C for 10 min			

Abbreviations: HE, heterozygote; MH, mutant homozygote; NH, normal homozygote.

### 2.5 Statistical Calculation

SPSS software package by IBM, version 23.0 (IBM, Armonk, New York, USA), was used for all statistical calculations. Chi‐square (*χ* ^2^) test, odds ratio (OR) along with their 95% confidence intervals (CI), and Hardy–Weinberg Equilibrium (HWE) were analyzed. The percentages for genotype and allelic frequency were provided. The characteristics of the included subjects were described by descriptive statistics using frequencies and percentages. We have analyzed the association of polymorphisms using six genetic models for each polymorphism such as additive model 1 (CT vs. CC), additive model 2 (TT vs. CC), dominant model (CT + TT vs. CC), recessive model (TT vs. CC + CT), overdominant model (CT vs. CC + TT), allele (T vs. C) models for *MMP2* rs2285053 and additive model 1 (AG vs. AA), additive model 2 (GG vs. AA), dominant model (AG + GG vs. AA), recessive model (GG vs. AA + AG), overdominant model (AG vs. AA + GG) and allele (G vs. A) models for *MMP9* rs3787268. A *p* value below 0.05 was considered to be statistically significant for all analyses.

## 3 Results

### 3.1 Distribution of Demographic Information Among Individuals

Among 105 breast cancer patients, most participants (52.38%) were < 45 years old, and 38.09% were aged between 45 and 60. The rest of the patients' ages were > 60 years. Most individuals from the control group were 45–60 years old, accounting for 50.00% of all controls. The age group < 45 and > 60 years represented 37.96% and 12.03%, respectively. From the demographic and clinicopathological parameters of the study participants in Table 3, it is evident that most patients had tumor stages II (70.48%). ER‐positive patients comprised 46.67% of the total, while ER‐negative patients accounted for 53.33%. According to PR status, 43.80% of patients had PR (+), and 56.19% had PR (−), whereas 44.76% of patients had HER2 (+) and 55.24% had HER2 (−).

**Table 3:** Distribution of demographic variables of breast cancer patients and controls.

Variables	Cases *n* = 105 (%)	Controls *n* = 108 (%)
Age (years)		
< 45	55 (52.38%)	41 (37.96%)
45–60	40 (38.09%)	54 (50.00%)
> 60	10 (9.52%)	13 (12.03%)
45–60 + > 60	54 (51.42%)	67 (62.03%)
Mean age (years)		
Minimum age	25	21
Maximum age	70	74
Average age	43.94	39.27
BMI (kg/m^2^)		
Average	29.39	22.30
Marital status		
Married	102 (97.14%)	90 (83.33%)
Unmarried	3 (2.85%)	18 (16.67%)
Histological types of breast cancer		
Atypical ductal hyperplasia	1 (0.09%)	N/A
Duct cell carcinoma	3 (2.85%)	N/A
Infiltrating duct cell carcinoma	34 (32.38%)	N/A
Intraductal carcinoma	1 (0.09%)	N/A
Invasive duct cell carcinoma	62 (59.05%)	N/A
Metastatic duct cell carcinoma	3 (2.85%)	N/A
Triple‐negative breast cancer	1 (0.09%)	N/A
TNM staging system		
Tumor size		
Tx	0	N/A
Tis	0	N/A
T0	26 (24.76%)	N/A
T1	30 (28.57%)	N/A
T2	34 (32.38%)	N/A
T3	13 (12.38%)	N/A
T4	2 (1.90%)	N/A
Nodal status		
Nx	23 (21.90%)	N/A
N0	19 (18.09%)	N/A
N1	41 (39.05%)	N/A
N2	15 (14.29%)	N/A
N3	7 (6.67%)	N/A
Distant metastasis		
M0	90 (85.71%)	N/A
M1	12 (11.43%)	N/A
Grade of breast cancer		
Ⅰ	20 (19.05%)	N/A
Ⅱ	74 (70.48%)	N/A
Ⅲ	21 (20.00%)	N/A
ER status		
ER (+)	49 (46.67%)	N/A
ER (−)	56 (53.33%)	N/A
PR status		
PR (+)	46 (43.80%)	N/A
PR (−)	59 (56.19%)	N/A
HER2 status		
HER2 (+)	47 (44.76%)	N/A
HER2 (−)	58 (55.24%)	N/A

### 3.2 Genotype Data Distribution Between Cases and Controls

Table 4 shows the distribution of HWE for the rs2285053 and rs3787268 polymorphisms of *MMP2* and *MMP9* genes, respectively. The genotype and allele frequencies of both SNPs differed significantly between the patients and the controls (*p* < 0.001). For the rs2285053 SNP, although 75% of breast cancer patients and 87% of healthy controls had the CC genotype, this percentage was only 23% and 12.96% for those with CT and 0.95% and 0.0% for those with TT genotypes, respectively. Both controls and cases were found to be consistent, according to the HWE test (*χ* ^2^ = 0.411, *p* = 0.522 and *χ* ^2^ = 0.519, *p* = 0.471). According to SNP genotype rs3787268 genotypes, 11.43% of patients and 2.78% of controls had AA genotypes, respectively. In cases, the percentages of AG and GG genotypes were 27.62% and 60.95%, respectively, while in controls, the numbers were 33.33% and 63.89%. For controls, the genotype distribution did not deviate from HWE (*χ* ^2^ = 3.64, *p* = 0.056, whereas for patients, the genotype distribution did not follow HWE (*χ* ^2^ = 7.55, *p* = 0.006).

**Table 4:** Genotype data distribution and Hardy–Weinberg equilibrium (HWE) test status.

				HWE			
				Cases		Controls	
SNP ID	Genotype/allele	Case *n* = 105 (%)	Control *n* = 108 (%)	*χ* ^2^	*p*	*χ* ^2^	*p*
rs2285053	CC	79 (75.24%)	94 (87.04%)				
	CT	25 (23.81%)	14 (12.96%)	0.411	0.522	0.519	0.471
	TT	1 (0.95%)	0 (0.00%)				
rs3787268	AA	12 (11.43%)	3 (2.78%)				
	AG	29 (27.62%)	36 (33.33%)	7.55	0.006	0.443	0.506
	GG	64 (60.95%)	69 (63.89%)				

### 3.3 Association of MMP2 rs2285053 and MMP9 rs3787268 With Breast Cancer

Table 5 describes the relationship between the rs2285053 variant and the risk of breast cancer. For females with the CT genotype, the chance of developing breast cancer was shown to be significantly higher than in those with the CC genotype (OR = 2.12, 95% CI = 1.03–4.36, *p* = 0.040). A higher risk of developing breast cancer (OR = 2.21, 95% CI = 1.08–4.52, *p* = 0.015) was also shown to be associated with the dominant model (CT + TT vs. CC). In the case of the overdominant model, we found a statistically significant relationship (CT vs. CC + TT: OR = 2.10, 95% CI = 1.02–4.31, *p* = 0.043). Subsequently, a carrier with the T allele showed 2.13 times of developing breast cancer compared to a carrier of the C allele (T vs. C: OR = 2.13, 95% CI = 1.08–4.18, *p* = 0.028), and this association was also statistically significant.

**Table 5:** Quantitative risk analysis of rs2285053 and rs3787268 polymorphism on breast cancer patients.

SNPs	Genetic model	Allele	Case (%)	Control (%)	Crude analysis		
					OR	95% CI	*p*
		CC	79 (75.24%)	94 (87.04%)	1		
	Additive model 1 (CT vs. CC)	CT	25 (23.81%)	14 (12.96%)	2.12	1.03–4.36	**0.040**
	Additive model 2 (TT vs. CC)	TT	1 (0.95%)	0 (0.00%)	3.57	0.14–88.76	0.439
	Dominant model (CT + TT vs. CC)	CC	79 (75.24%)	94 (87.04%)	1		
		CT + TT	26 (24.76%)	14 (12.96%)	2.21	1.08–4.52	**0.015**
rs2285053	Recessive model (TT vs. CC + CT)	CC + CT	104 (99.05%)	108 (100.00%)	1		
		TT	1 (0.95%)	0 (0.00%)	3.11	0.12–77.33	0.491
	Overdominant model (CT vs. CC + TT)	CC + TT	80 (76.19%)	94 (87.04%)	1		
		CT	25 (23.81%)	14 (12.96%)	2.10	1.02–4.31	**0.043**
	Allele (T vs. C)	C	183 (87.14%)	202 (93.52%)	1		
		T	27 (12.86%)	14 (6.48%)	2.13	1.08–4.18	**0.028**
		AA	12 (11.43%)	3 (2.78%)	1		
	Additive model 1 (AG vs. AA)	AG	29 (27.62%)	36 (33.33%)	0.20	0.05–0.78	**0.021**
	Additive model 2 (GG vs. AA)	GG	64 (60.95%)	69 (63.89%)	0.23	0.06–0.86	**0.029**
rs3787268	Dominant model (AG + GG vs. AA)	AA	12 (11.43%)	3 (2.78%)	1		
		AG + GG	93 (88.57%)	105 (97.22%)	0.22	0.06–0.81	**0.022**
	Recessive model (GG vs. AA + AG)	AA + AG	41 (39.05%)	39 (36.11%)	1		
		GG	64 (60.95%)	69 (63.89%)	0.88	0.51–1.54	0.661
	Overdominant model (AG vs. AA + GG)	AA + GG	76 (72.38%)	72 (66.67%)	1		
		AG	29 (27.62%)	36 (33.33%)	0.76	0.42–1.37	0.372
	Allele (G vs. A)	A	53 (25.24%)	42 (19.44%)	1		
		G	157 (74.76%)	174 (80.56%)	0.71	0.45–1.13	0.154

*Note:* In the case of the test of association, bold indicates statistically significant (*p* < 0.05).

In the case of the rs3787268 variant, three genetic models were associated with decreased risk of breast cancer, and the results were statistically significant (additive model 1—AG vs. AA: OR = 0.20, 95% CI = 0.05–0.78, *p* = 0.021; additive model 2—GG vs. AA: OR = 0.23, 95% CI = 0.06–0.86, *p* = 0.029; dominant model—AG + GG vs. AA: OR = 0.22, 95% CI = 0.06–0.81, *p* = 0.023). This result indicates that the rs3787268 polymorphism shows a protective effect against breast cancer (Table 5).

## 4 Discussion

Each year, millions of individuals lose their lives due to breast cancer, making it the leading cause of cancer‐related death worldwide [34]. Factors like low parity, genetic history of breast cancer, premature menarche and late menopause, hormone replacement therapy, and postmenopausal obesity are all known to increase the likelihood of developing breast cancer [35]. Testing for *BRCA1* and *BRCA2* mutations has become standard practice for females with breast cancer in their families, but other genetic abnormalities or variations may also be relevant in therapeutic settings [36]. Over 90% of breast cancer patients could experience an increase in their life expectancy with early detection and effective treatment [37]. The survival percentage for females diagnosed with breast cancer can be significantly improved through early identification if more people in low‐resource nations like Bangladesh are made aware of the disease [38].

MMPs are endopeptidases with crucial functions in cancer development and subsequent spread throughout the body [39]. MMP2 (gelatinase A) and MMP9 (gelatinase B) are members of the MMPs family that have been shown to play a functional role in tumor angiogenesis, invasion, and metastasis, as well as in the carcinogenesis of breast cancer [19, 40]. However, research into the putative correlations between MMP2 or MMP9 expression, clinicopathological features, and survival in breast cancer has yielded conflicting results. Tumor metastasis is an important event in breast cancer that substantially impacts patient survival and can change treatment options. Overproduction of MMP2 or MMP9 leads to the breakdown of critical ECM and BM components, allowing tumor cells to escape and spread further [41]. MMP2 and MMP9, which catalyze the breakdown of gelatin IV, the primary component of the extracellular matrix, are the most common MMPs found in breast cancer. *MMP2* and *MMP9* have thus been potentially linked to breast cancer cell invasion and metastasis [42, 43].

As is observed, the present study reported a significant association of *MMP2* rs2285053 polymorphism with breast cancer risk in four genetic models, including the additive model 1, the dominant model, the overdominant model, and the allele model. To date, only three studies have investigated the association between rs2285053 in *MMP2* and breast cancer risk. According to bioinformatics investigations, the *MMP2* variant rs2285053 has the potential to modify a Sp1 binding site and hence affect MMP2 transcription, as reported by Yu et al. [44]. According to two previous studies, breast cancer risk was shown to be lower in Tunisians [45] and Iranians [46] who had the rs2285053 T allele rather than the C allele. On the contrary, Beeghly‐Fadiel and colleagues reported no link between rs2285053 polymorphisms and breast cancer risk in Chinese females [47]. This discrepancy in the results could be attributed to ethnic differences in the population.

On the other hand, our study suggests that *MMP9* rs3787268 shows a protective effect on breast cancer susceptibility in three genetic models, including the additive model 1, the additive model 2, and the dominant model. A previous case‐control study on 251 breast cancer patients and 255 controls conducted by Fu et al. [29] suggested that this polymorphism was not associated with breast cancer. Slattery and colleagues suggested that Native American females are more likely to develop breast cancer if they carry the G to A variant of *MMP9* rs3787268 and found that the GA and AA genotypes of rs3787268 were significantly associated with a 1.52‐fold risk of breast cancer in the same population [31]. However, in our study, we found that three genetic models of rs3787268 were associated with decreased risk of breast cancer in Bangladeshi females, and our study is consistent with Wang and colleagues, who suggested that the minor allele (A) of rs3787268 was associated with decreased risk of breast cancer in Chinese females [30]. Moreover, two meta‐analyses also found no association of breast cancer with this *MMP9* rs3787268 polymorphism [48, 49].

## 5 Conclusion

The current study concludes that *MMP2* (rs2285053) is associated with an increased risk of breast cancer, while *MMP9* (rs3787268) polymorphisms may be correlated with a reduced risk of breast cancer in Bangladeshi females. However, due to the small sample size in the current study, large‐scale and cross‐population studies are needed to validate the results of the association between these polymorphisms and breast cancer risk.

## Author Contributions

**Pulak Chowdhury:** investigation, validation, methodology, visualization, writing – original draft, writing – review and editing, formal analysis, data curation, software. **Md. Abdul Aziz:** investigation, writing – original draft, validation, methodology, visualization, writing – review and editing, formal analysis, software, data curation. **Tahmina Akter:** investigation, writing – original draft, validation, methodology, data curation. **Mohammad Safiqul Islam:** conceptualization, validation, methodology, visualization, writing – review and editing, project administration, resources, supervision. **Md. Shahid Sarwar:** conceptualization, writing – review and editing, visualization, methodology, validation, software, project administration, supervision, resources.

## Consent

Informed consent was obtained from the participants before conducting the study.

## Conflicts of Interest

Dr. Mohammad Safiqul Islam is an Editorial Board member of Health Science Reports and a coauthor of this article. To minimize bias, he was excluded from all editorial decision‐making related to the acceptance of this article for publication.

## Transparency Statement

The lead author Md. Shahid Sarwar affirms that this manuscript is an honest, accurate, and transparent account of the study being reported; that no important aspects of the study have been omitted; and that any discrepancies from the study as planned (and, if relevant, registered) have been explained.

## Data Availability

The data that support the findings of this study are available from the corresponding author upon reasonable request.
